# Brazilian dietary patterns and the dietary approaches to stop hypertension (DASH) diet-relationship with metabolic syndrome and newly diagnosed diabetes in the ELSA-Brasil study

**DOI:** 10.1186/s13098-017-0211-7

**Published:** 2017-02-13

**Authors:** Michele Drehmer, Andrew O. Odegaard, Maria Inês Schmidt, Bruce B. Duncan, Letícia de Oliveira Cardoso, Sheila M. Alvim Matos, Maria del Carmen B. Molina, Sandhi M. Barreto, Mark A. Pereira

**Affiliations:** 1 Department of Nutrition, School of Medicine, Federal University of Rio Grande do Sul, Rua Ramiro Barcelos, 2400, 4o andar, Porto Alegre, RS Brazil; 2 Postgraduate Program in Epidemiology and Hospital de Clínicas de Porto Alegre, School of Medicine, Federal University of Rio Grande do Sul, Rua Ramiro Barcelos 2600, sala 419, Porto Alegre, RS Brazil; 3 Department of Epidemiology, School of Medicine, University of California, Irvine, USA; 4 Department of Social Medicine, School of Medicine, Federal University of Rio Grande do Sul, Rua Ramiro Barcelos, 2600, sala 419, Porto Alegre, RS Brazil; 5 National School of Public Health Sergio Arouca, Fundação Oswaldo Cruz, Rio de Janeiro, Brazil; 6 Institute of Collective Health, Federal University of Bahia, 513 Araújo Pinho Ave., Salvador, Brazil; 7 Health Science Center, Federal University of Espírito Santo, Vitória, Brazil; 8 Research Group on Epidemiology on Chronic and Occupational Diseases (GERMINAL), School of Medicine, Universidade Federal de Minas Gerais, Belo Horizonte, Minas Gerais 30130-100 Brazil; 9 Division of Epidemiology and Community Health, School of Public Health, University of Minnesota, 1300 S. 2nd Street, Suite 300, Minneapolis, MN USA

**Keywords:** Dietary patterns, Diabetes, Metabolic syndrome, Cohort study

## Abstract

**Background:**

Studies evaluating dietary patterns, including the DASH diet, and their relationship with the metabolic syndrome and diabetes may help to understand the role of dairy products (low fat or full fat) in these conditions. Our aim is to identify dietary patterns in Brazilian adults and compare them with the (DASH) diet quality score in terms of their associations with metabolic syndrome and newly diagnosed diabetes in the Brazilian Longitudinal Study of Adult Health-the ELSA-Brasil study.

**Methods:**

The ELSA-Brasil is a multicenter cohort study comprising 15,105 civil servants, aged 35–74 years at baseline (2008–2010). Standardized interviews and exams were carried out, including an OGTT. We analyzed baseline data for 10,010 subjects. Dietary patterns were derived by principal component analysis. Multivariable logistic regression investigated associations of dietary patterns with metabolic syndrome and newly diagnosed diabetes and multivariable linear regression with components of metabolic syndrome.

**Results:**

After controlling for potential confounders, we observed that greater adherence to the Common Brazilian meal pattern (white rice, beans, beer, processed and fresh meats), was associated with higher frequencies of newly diagnosed diabetes, metabolic syndrome and all of its components, except HDL-C. Participants with greater intake of a Common Brazilian fast foods/full fat dairy/milk based desserts pattern presented less newly diagnosed diabetes. An inverse association was also seen between the DASH Diet pattern and the metabolic syndrome, blood pressure and waist circumference. Diet, light foods and beverages/low fat dairy pattern was associated with more prevalence of both outcomes, and higher fasting glucose, HDL-C, waist circumference (among men) and lower blood pressure. Vegetables/fruit dietary pattern did not protect against metabolic syndrome and newly diagnosed diabetes but was associated with lower waist circumference.

**Conclusions:**

The inverse associations found for the dietary pattern characterizing Brazilian fast foods and desserts, typically containing dairy products, with newly diagnosed diabetes, and for the DASH diet with metabolic syndrome, support previously demonstrated beneficial effects of dairy products in metabolism. The positive association with metabolic syndrome and newly diagnosed diabetes found for the pattern characterizing a typical Brazilian meal deserves further investigation, particularly since it is frequently accompanied by processed meat.

*Trial registration* NCT02320461. Registered 18 December 2014

**Electronic supplementary material:**

The online version of this article (doi:10.1186/s13098-017-0211-7) contains supplementary material, which is available to authorized users.

## Background

Type 2 diabetes and metabolic syndrome are global public health problems [1, 2], and nutritional interventions have been recommended for their prevention and control [3–5]. Studies investigating dietary patterns provide valuable information to assess healthful or harmful dietary habits, beyond those analyzing individual nutrients or food groups alone [6–8], since foods are consumed in complex combinations and nutrients may have interactive effects [9, 10].

A recent meta-analysis of prospective observational studies including 404,528 individuals, revealed that adherence to ‘healthy’ dietary patterns significantly reduced the risk of diabetes (RR = 0.86; 95% CI 0.82, 0.90), while ‘unhealthy’ dietary patterns (generally emphasizing red meat and refined carbohydrate based foods) increased risk (RR = 1.30; 95% CI 1.18, 1.43) [11]. Additionally, a Western dietary pattern, with strong components of refined carbohydrates, and red and processed meats, is associated with metabolic syndrome and cardiovascular diseases (CVD) [12–14].

The dietary approaches to stop hypertension (DASH) diet, a well-known dietary pattern specifically targeted to lowering blood pressure, was associated with lower CVD risk in US [15, 16] and European populations [17]. The DASH diet also has the potential to prevent type 2 diabetes and stroke [18, 19]. A meta-analysis of intervention studies based on the DASH diet showed reduced fasting insulin concentration and improved insulin sensitivity independently of weight loss [20]. The DASH diet emphasizes high intake of fruits, vegetables, nuts, legumes, whole grains, low-fat dairy products as well as a low intake of sodium, red and processed meats, and sweetened beverages.

The specific benefits of an increased intake of dairy foods have recently received greater attention. A pooled analysis of 7 cohort studies (254,892 participants and 19,082 cases of diabetes) revealed that higher intake of total dairy products was associated with lower risk of type 2 diabetes. Similar inverse associations were found for low-fat dairy products, low-fat or skim milk and cheese, and for yogurt, but not for high-fat dairy products or total milk; results regarding the metabolic syndrome were inconclusive [21]. Additionally, biomarkers of dairy intake are related to a decreased incidence of diabetes [22]. Analyzing data from the Brazilian Longitudinal Study of Adult Health (ELSA-Brasil) study, we found that the intake of total dairy products was inversely associated with measures of glycemia, insulinemia and metabolic syndrome with a linear dose–response pattern. Interestingly, these associations were observed for full-fat dairy products and yogurt but not for low-fat dairy products [23, 24].

Further investigation of dietary patterns and their relationship with diabetes and the metabolic syndrome in distinct populations may provide valuable information with regard to the role of specific combinations of foods and nutrients. Yet, studies evaluating dietary patterns, including the DASH diet, in relation to metabolic syndrome and diabetes are scarce, especially in populations outside of Europe and North America [25]. The present study aims to identify dietary patterns in Brazilian adults and compare their associations, as well as those of the (DASH) diet quality score, with metabolic syndrome and newly diagnosed diabetes.

## Methods

### Study design

The Brazilian Longitudinal Study of Adult Health is a multicenter cohort study and comprises 15,105 civil servant volunteers, aged 35–74 years at baseline (2008–2010), from universities or research institutions located in six Brazilian capitals (Belo Horizonte, Porto Alegre, Rio de Janeiro, Salvador, São Paulo, Vitoria). Description of study design and sample characteristics have been previously described [26]. The study was approved by the local research and ethics committees of participating institutions, and all participants provided written consent.

### Study participants

For the current investigation we used data from the baseline examination. We excluded participants with previously diagnosed diabetes (diabetes status reported during initial interviews, or taking oral hypoglycemic medications or insulin [n = 1473]), self-reported chronic disease as CVD [n = 1280], cancer [n = 695], and other chronic diseases [n = 2649] (stroke, emphysema, bronchitis, chronic obstructive pulmonary disease, cirrhosis, hepatitis, cardiac or bariatric surgery, rheumatic fever, Chagas disease, and thrombosis or emboli), and with unusually low (2nd percentile; ≤1298 kcal/day) or high (98th percentile, ≥6372 kcal/day) reported energy intake [n = 629], which left 10,010 subjects for analysis. For analyses that involves metabolic syndrome, we also excluded participants with a fasting state either <12 or >15 h [n = 542]. Some participants had more than 1 exclusion criteria, resulting in 9835 participants.

### Assessment of diet and covariates

Dietary data were collected using a validated food frequency questionnaire (FFQ) [27], with 114 food and drink items and covering the last 12 months. Participants were also asked to provide information on their typical eating habits, including attempts to modify their diet in the 6 months before the baseline examination.

We transformed the frequency options into daily frequencies as follows: 3 for more than 3 times/day; 2.5 for 2–3 times/day; 1 for once/day; 0.8 for 5–6 times/week; 0.4 for 2–4 times/week; 0.1 for once/week; 0.07 for 1–3 times/month; and 0 for never/almost never. Additionally, we obtained daily energy intake in kilocalories and nutrient intake using the University of Minnesota Nutrition Data System for Research database [28]. For these determinations, we calculated the grams/day for each food item from the FFQ as the quantity of servings consumed/day × weight (standard portion in grams) × frequency of consumption.

At baseline, trained interviewers collected demographic characteristics (age, sex, race, educational level, family income, occupational status, study site), family history of diabetes, menopausal status and lifestyle factors: smoking (current and previous), alcohol intake and physical activity. Alcohol intake was estimated as the sum of ethanol (g/week) of all beverages consumed. Physical activity variables were defined by using the leisure activity section of the International Physical Activity Questionnaire (IPAQ), long form, according to the IPAQ guidelines for data processing and analysis. Median (interquartile range) metabolic equivalent min/week were computed for walking, moderate-intensity and vigorous-intensity activities, and summed to obtain a combined leisure-time total physical activity score [29].

We performed anthropometric measurements (weight, height, and waist circumference) with participants standing, dressed in light standard uniforms, without shoes in the fasting state. We measured body weight to the nearest 0.1 kg with a calibrated balance (Toledo 2096PP) and height with a vertical stadiometer (Seca-SE-216) to the nearest 0.1 cm. Waist circumference was measured with a tape measure to the nearest 0.1 cm around the midpoint between the inferior costal border and the iliac crest. BMI was calculated as weight (kg) divided by height squared (m^2^).

### Definition of metabolic syndrome

Blood was drawn by venipuncture after an overnight fast of 8–15 h, and a standard 2-h 75-g oral glucose tolerance test was administered for all participants who did not report a diagnosis or current treatment for diabetes. Glucose was measured by an ADVIA 1200 chemistry hexokinase system (Siemens); glycated hemoglobin using an HPLC assay (Bio-Rad D-10 Dual Program Laboratories) certified by the National Glycohemoglobin Standardization Program; and HDL-cholesterol and triglycerides using enzymatic procedures (ADVIA 1200).

Resting blood pressure was measured 3 times at 1-min intervals using a 765CP oscillometric sphygmomanometer (Omron) with participants seated after a 5-min rest. The average of the second and third measurements was used in the analyses.

We used the joint interim statement consensus criteria [30] for diagnosing metabolic syndrome, which requires the presence of any 3 of the following 5 risk factors: elevated waist circumference (≥102 cm in men and ≥88 cm in women), hypertriglyceridemia (≥1.69 mmol/L/150 mg/dL or drug treatment), reduced HDL-cholesterol (<1.03 mmol/L/<40 mg/dL for men and <1.29 mmol/L/<50 mg/dL for women, or drug treatment), elevated blood pressure (≥130 mmHg; diastolic blood pressure ≥85 mmHg and/or drug treatment), and elevated fasting glucose (≥100 mg/dL/5.6 mmol/L or drug treatment).

### Definition of newly diagnosed diabetes

After excluding participants with previously diagnosed diabetes, we considered as newly diagnosed diabetes participants with fasting glucose ≥126 mg/dL, 2-h postload glucose ≥200 mg/dL or HbA1c ≥6.5%.

### Statistical analysis

Dietary patterns were derived using principal component analysis (PCA). All 114 foods and beverages, including alcohol, were standardized to per day units. The Kaiser–Meyer–Olkin test (>0.6) and Bartlett’s test of sphericity (p < 0.05) were applied to verify whether the PCA assumptions were all met. The factors were rotated orthogonally by the varimax method, and those with eigenvalues ≥1.5 were retained. To determine the number of factors to retain, we also considered the scree plot and factor interpretability. Factor was interpreted based on food items with loadings ≥0.20. We identified four dietary patterns and named them according to food items showing high loadings (Table 1). Factors extracted did not vary by sex and study site. Factor scores were determined for each participant by multiplying standardized intake of each food item (grams/day) by its respective loading factor on each factor. Factor scores were thus linear variables, representing the weighted sum across all 114 food and beverage items. Individuals were classified into quintiles of each factor score. Results are presented for three groups: high adherence (fifth quintile); mid adherence (third quintile); low adherence (first quintile) to a given pattern quintile.

A DASH Diet quality score was also created to assess adherence to the DASH diet [16]. This score considered intake of fruits, vegetables, nuts and legumes, whole grains, low-fat dairy products as well as that of sodium, red and processed meats, and sweetened beverages. For each of these components, subjects were classified into sex-specific quintiles according to their intake, with values being assigned to each quintile (from 1 for the lowest intake to 5 for the highest, except for the components sodium, and red and processed meats, and sweetened beverages for which the scoring was reversed). We summed up the eight DASH component scores to obtain an overall DASH score ranging from 8 (lowest adherence) to 40 (highest adherence) [17].

Multivariable logistic regression was used to evaluate the adjusted association of the different dietary patterns with metabolic syndrome and newly diagnosed diabetes. A linear trend was tested by modeling categorical quintiles of dietary patterns consumption as a continuous variable in the multivariable regression models. We evaluated interactions between dietary patterns and excess weight (BMI ≥25 kg/m^2^). We adjusted for covariates (potential confounders) in 2 models: model 1 included demographic covariates, menopausal status, and family history of diabetes; model 2 included all variables from model 1 plus body mass index (BMI), physical activity, current and previous smoking, alcohol intake, and calories/day. Our intention was to separate the adjustment for obesity and related behavioral characteristics. Since obesity may be interpreted as a confounder or as a mediator of the associations, we provide results for both models, facilitating interpretation of potential confounding. Multivariable linear regression was performed to investigate adjusted associations of different dietary patterns with components of metabolic syndrome: fasting glucose, triglycerides, HDL cholesterol, waist circumference, systolic and diastolic blood pressure. We adjusted for covariates using similar models.

All analyses were performed in the statistical package SPSS version 18 (IBM).

## Results

The mean ± SD age was 50.7 ± 8.7 year, and 54.8% (n = 5390) were women. Four main dietary patterns were derived from PCA (Table 1). The first pattern, “vegetables/fruits” (P1), had high loadings for vegetables and fruits (not including fruit juice). The second pattern, “Common Brazilian fast foods/full fat dairy/desserts” (P2), had high loadings for fast foods (fried or baked), cakes, milk based desserts, regular cheese and red meats (fresh and processed), which characterizes, in general a common pattern of eating in fast food snack bars/restaurants in Brazil. The third pattern, “Common Brazilian meal” (P3), had high loadings for white rice, beans, beer, processed and fresh meats, which includes the classic Brazilian meal (black beans, rice and meat). The fourth pattern “Diet or light foods and beverages/low fat dairy” (P4) had high loadings for low fat foods, low or zero sugar beverages with artificial sweeteners and low fat dairy, which characterizes a pattern of eating of those interested in improving their health by eating less calories/less sugar or by medical counseling. Mean servings/day for specific food groups according to the quintiles of dietary patterns scores are presented in Additional file 1: Table S1. Participant characteristics according to low, mid and high adherence to dietary patterns are presented in Additional file 1: Table S2. Those with higher vegetables/fruits (P1) scores were older, more often women, less frequently a university graduate, smoked less, drank less alcohol, were more active, and had lower intake of total fat and saturated fat, and greater consumption of carbohydrate. Those with higher Common Brazilian fast foods/full-fat dairy/desserts (P2) scores were predominantly men, younger, white, with a university degree, and had greater intake of total and saturated fat, and less protein consumption. Those with a higher Common Brazilian meal (P3) score were predominantly men, and on average slightly younger, of mixed color-race (“Pardo”), less frequently a university graduate, smoked more, drank more alcohol, were less active and had slightly higher BMI. Those with higher scores in the Diet, light foods and beverages/low fat dairy (P4) pattern were more frequently women, white, with a university degree, smoked less, drank more alcohol, were more active, with high protein consumption, less carbohydrate intake and higher BMI. Finally, those with higher adherence to DASH Diet were more frequently women, elderly, white, with a university degree, smoked less, drank less alcohol, were more active and had a lower BMI. They had less total fat and saturated fat consumption and more carbohydrate intake.

**Table 1:** Dietary patterns extracted using principal component analysis (n = 9835)

Dietary Patterns (P1–P4)	Loading weights ≥0.4	Loading weights 0.4–0.3	Cumulative % of the variance explained
Vegetables/fruits (P1)	Zucchini/chayote/eggplant, collard greens, carrots, green beans, cabbage, lettuce, chicory/watercress/arugula, tomato	Orange/tangerine, pumpkin, apple/pear, beets, cauliflower, broccoli, papaya, banana, okra, pineapple, onion, melon, grape	4.3
Common Brazilian fast foods/full fat dairy/desserts (P2)	Pizza, chocolate, fries, flan/mousses	Ham/salami, fried fast foods, baked fast foods, layered cake, beef stroganoff, ice cream, hot dog, mozzarella and other yellow cheeses, hamburger patty, regular soda	8.4
Common Brazilian meal (P3)	White rice, beans, garlic	Beer, sausage, tripe/stomach, bacon, onion, fried chicken, pork	11.0
Diet, light foods and beverages/low fat dairy (P4)	Low fat turkey breast	Low fat yogurt, coffee with artificial sweetener, diet, light or zero soda, low calorie bread, low fat cream cheese	13.1

Extraction method: principal component analysis using varimax rotation

The frequency of metabolic syndrome was 30.7% (n = 3019) and of newly diagnosed diabetes, 10.9% (n = 1044). Table 2 presents unadjusted frequencies of metabolic syndrome and newly diagnosed diabetes according to levels of adherence (low, mid and high) to dietary patterns. Frequency of metabolic syndrome increased with levels of adherence to the Common Brazilian meal and the Diet, light foods and beverages/low fat dairy dietary patterns, but decreased with higher adherence to the DASH Diet. Frequency of newly diagnosed diabetes increased with levels of adherence to the Common Brazilian meal (frequency was twice that of low adherence). In contrast, lower frequency of newly diagnosed diabetes was seen in those of high adherence to the Common Brazilian fast foods/full fat dairy/desserts pattern.

**Table 2:** Metabolic syndrome and newly diagnosed diabetes by adherence to dietary patterns (n = 9835)

Outcomes	Dietary patterns (P1–P4)	Adherence to different dietary patterns (in quintiles)		
		Quintile 1—low adherence	Quintile 3—mid adherence	Quintile 5—high adherence
		N (%)		
Metabolic syndrome, n (%)	P1—Vegetables/fruits	594 (30.6)	558 (28.7)	677 (34.9)
	P2—Common Brazilian fast food/Full fat dairy/desserts	704 (36.2)	545 (28.0)	714 (36.7)
	P3—Common Brazilian meal	421 (21.6)	591 (30.4)	786 (40.4)
	P4—Diet, light foods and beverages/low fat dairy	540 (27.8)	616 (31.7)	629 (32.4)
	DASH diet	571 (32.7)	712 (31.2)	538 (29.4)
Newly diagnosed diabetes, n (%)	P1—Vegetables/Fruits	184 (9.5)	189 (9.7)	261 (13.4)
	P2—Common Brazilian fast food/Full fat dairy/desserts	314 (16.1)	183 (9.4)	182 (9.4)
	P3—Common Brazilian	128 (6.6)	185 (9.5)	314 (16.1)
	P4—Diet, light foods and beverages/low fat dairy	209 (10.7)	197 (10.1)	199 (10.2)
	DASH diet	176 (10.1)	247 (10.8)	200 (10.9)

Table 3 shows adjusted odds ratios for metabolic syndrome and newly diagnosed diabetes according to the adherence to each pattern when categorized in quintiles. Strong and graded positive association remained for the Common Brazilian meal pattern for both metabolic syndrome and newly diagnosed diabetes after adjustment (model 2), with odds of presenting either outcome being approximately twice as frequent in the highest compared to the lowest quintile. Similarly, for the Diet, light foods and beverages/low fat dairy pattern, we also found positive associations for both outcomes, those in the 5th quintile, compared to the 1st quintile having approximately 50% greater odds of presenting either outcome.

**Table 3:** Adjusted associations of metabolic syndrome and newly diagnosed diabetes with adherence to dietary patterns (n = 9835)

	Metabolic syndrome criteria^a^ (OR and 95% CI)					P for trend^b^
	Q1	Q2	Q3	Q4	Q5	
P1—Vegetables/fruits						
Model 1	1.00	0.87 (0.75, 1.00)	0.87 (0.75, 1.00)	0.96 (0.83, 1.10)	1.04 (0.90, 1.20)	0.229
Model 2	1.00	0.91 (0.77, 1.06)	0.88 (0.75, 1.03)	0.96 (0.82, 1.13)	1.07 (0.90, 1.28)	0.366
P2—Common Brazilian fast foods/full fat dairy/milk based desserts						
Model 1	1.00	0.85 (0.74, 0.98)	0.86 (0.74, 0.99)	0.89 (0.77, 1.03)	1.08 (0.94, 1.25)	0.225
Model 2	1.00	0.83 (0.71, 0.97)	0.81 (0.69, 0.95)	0.77 (0.65, 0.91)	0.86 (0.71, 1.04)	0.057
P3—Common Brazilian meal						
Model 1	1.00	1.51 (1.29, 1.75)	1.81 (1.55, 2.11)	2.26 (1.93, 2.65)	2.89 (2.45, 3.42)	<0.001
Model 2	1.00	1.32 (1.12, 1.56)	1.48 (1.25, 1.76)	1.81 (1.52, 2.15)	2.13 (1.76, 2.58)	<0.001
P4—Diet, light foods and beverages/low fat dairy						
Model 1	1.00	1.31 (1.13, 1.51)	1.59 (1.37, 1.85)	1.78 (1.53, 2.08)	2.02 (1.72, 2.38)	<0.001
Model 2	1.00	1.24 (1.05, 1.46)	1.40 (1.18, 1.65)	1.44 (1.21, 1.71)	1.47 (1.23, 1.71)	<0.001
DASH diet pattern						
Model 1	1.00	0.90 (0.78, 1.03)	0.88 (0.77, 1.01)	0.75 (0.64, 0.86)	0.72 (0.61, 0.83)	<0.001
Model 2	1.00	0.94 (0.80, 1.10)	0.96 (0.82, 1.11)	0.81 (0.69, 0.95)	0.88 (0.74, 1.05)	0.044
Newly diagnosed diabetes^c^						
P1—Vegetables/fruits						
Model 1	1.00	1.01 (0.81, 1.25)	0.95 (0.76, 1.19)	0.99 (0.80, 1.24)	1.19 (0.96, 1.47)	0.132
Model 2	1.00	1.05 (0.84, 1.32)	1.00 (0.79, 1.26)	1.04 (0.82, 1.31)	1.29 (1.01, 1.65)	0.077
P2—Common Brazilian fast foods/Full fat dairy/milk based desserts						
Model 1	1.00	0.70 (0.57, 0.85)	0.71 (0.57, 0.87)	0.66 (0.53, 0.82)	0.76 (0.61, 0.94)	0.007
Model 2	1.00	0.67 (0.54, 0.82)	0.66 (0.53, 0.81)	0.57 (0.45, 0.72)	0.59 (0.46, 0.77)	<0.001
P3—Common Brazilian meal						
Model 1	1.00	1.54 (1.21, 1.97)	1.58 (1.23, 2.02)	1.92 (1.50, 2.46)	2.53 (1.96, 3.25)	<0.001
Model 2	1.00	1.41 (1.09, 1.80)	1.42 (1.10, 1.82)	1.64 (1.28, 2.12)	2.04 (1.56, 2.68)	<0.001
P4—Diet, light foods and beverages/low fat dairy						
Model 1	1.00	1.23 (0.99, 1.52)	1.29 (1.04, 1.61)	1.61 (1.28, 2.01)	1.71 (1.35, 2.18)	<0.001
Model 2	1.00	1.19 (0.96, 1.48)	1.21 (0.96, 1.52)	1.46 (1.15, 1.84)	1.45 (1.13, 1.88)	0.001
DASH Diet Pattern						
Model 1	1.00	1.01 (0.82, 1.26)	1.01 (0.82, 1.25)	0.94 (0.75, 1.18)	0.91 (0.72, 1.15)	0.324
Model 2	1.00	1.04 (0.83, 1.29)	1.07 (0.86, 1.32)	1.01 (0.80, 1.27)	1.07 (0.84, 1.36)	0.702

*Model 1* Adjusted through multiple logistic regression for the following variables: age (y), sex, race, education, family income (amount), occupational status, study center, menopausal status and family history of diabetes

*Model 2* Model 1 + BMI (kg/m^2^), physical activity (metabolic equivalent min/week), current and previous smoking status, alcohol (grams of ethanol/day) and calorie intake (kcal/day)

^a^Defined by the Joint Interim Statement Consensus Criteria, 2009 (see “Methods”)

^b^PCA components entered as continuous scores

^c^Defined by fasting glucose, oral glucose tolerance test or glycated hemoglobin

In contrast, as seen also in Table 3, participants who adhered to the Common Brazilian fast foods/full fat dairy/desserts dietary pattern had a considerably lower prevalence of newly diagnosed diabetes (OR for quintile 5 vs. quintile 1 = 0.59; 0.50–0.86) and a borderline statistically significant lower prevalence of metabolic syndrome. A borderline inverse association was seen also between higher adherence to the DASH Diet pattern and metabolic syndrome. When we adjusted additionally for full fat dairy, greater adherence to the Common Brazilian fast foods/full-fat dairy/desserts pattern was no longer associated with the metabolic syndrome, and the association with new onset diabetes moved slightly towards the null. Those with a greater vegetables/fruits pattern showed no association with the metabolic syndrome and inconsistently increased odds for having diabetes.

**Fig. 1:**
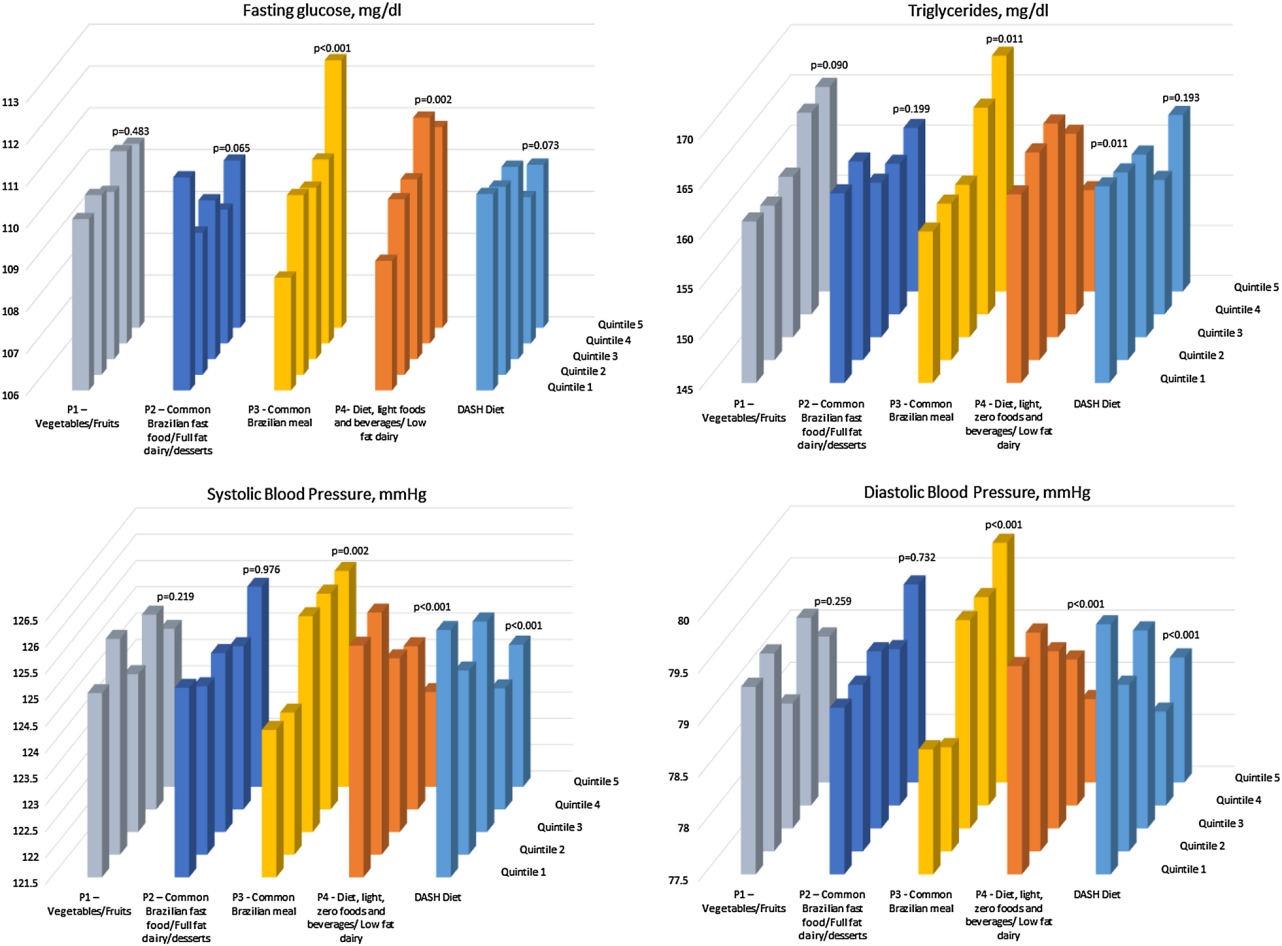
Adjusted* means for fasting glucose (mg/dL), triglycerides (mg/dL), systolic and diastolic blood pressure according to quintiles of adherence to the four dietary patterns identified and to the DASH-diet. ELSA-Brasil 2008–2010 (n = 9835). *Means obtained through multiple linear regression adjusting for: race, age, sex, education, study center, menopause, occupational status, family history of diabetes, BMI, physical activity, smoking, alcohol, calories/day

**Fig. 2:**
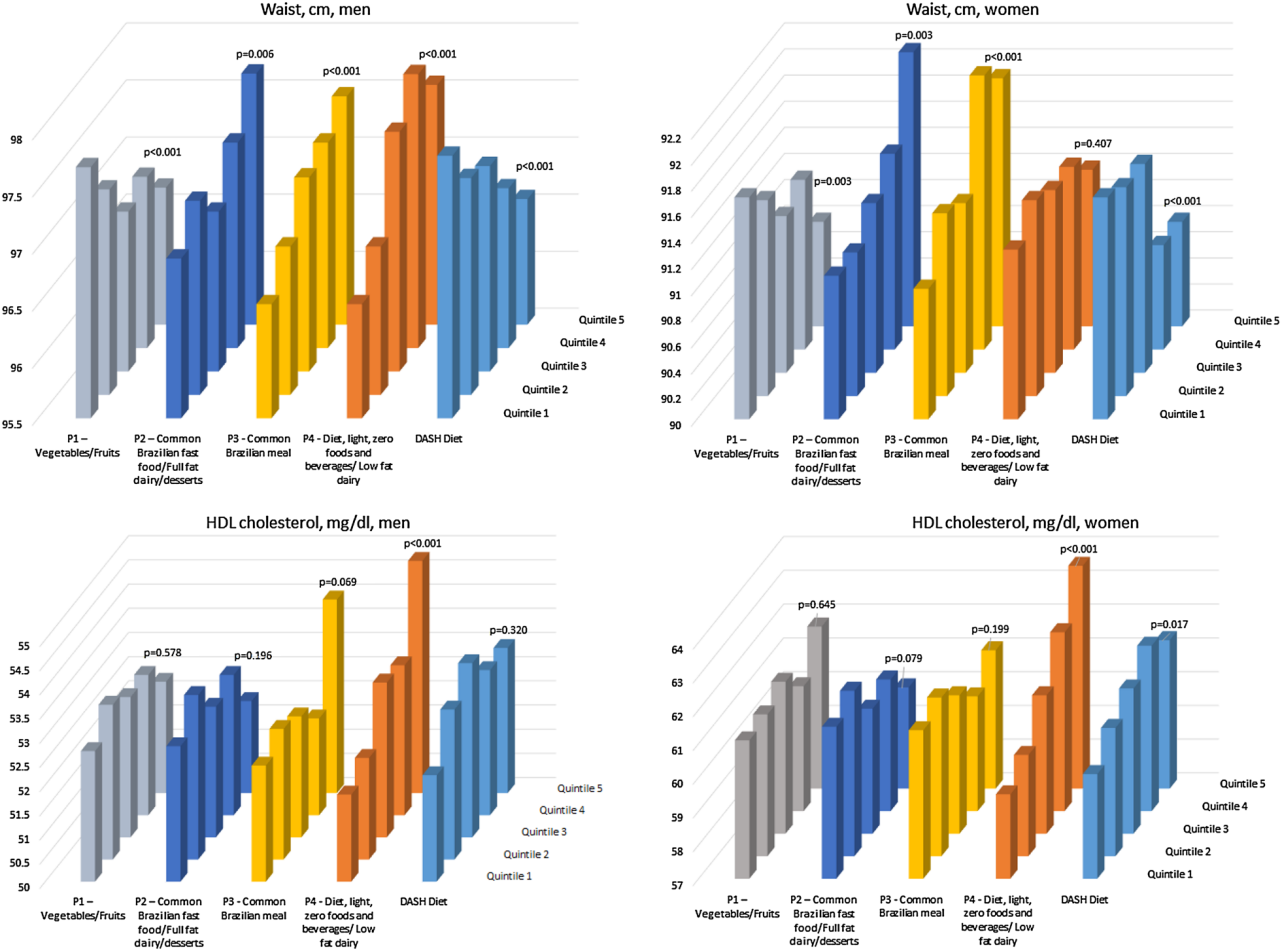
Adjusted* means for waist circumference (cm) in men and women and HDL cholesterol (mg/dL) in men and women according to quintiles of adherence to the four dietary patterns identified and to the DASH-diet. ELSA-Brasil 2008–2010 (n = 9835). *Means obtained through multiple linear regression adjusting for: race, age, sex, education, study center, menopause, occupational status, family history of diabetes, BMI, physical activity, smoking, alcohol, calories/day

Figures 1 and 2 present means of all components of metabolic syndrome according to adherence to dietary patterns and the DASH Diet, adjusted in similar models. We observed graded, positive associations between adherence to the Common Brazilian meal pattern and adjusted mean values for all syndrome components except HDL-cholesterol. Positive associations were also found for the diet, light foods and beverages/low fat dairy pattern with levels of fasting glucose, HDL-cholesterol and, in men only, for waist circumference. However, an inverse association for this dietary pattern was seen with systolic and diastolic blood pressure, similarly with the findings seen for the DASH diet. The vegetables/fruits pattern and the DASH diet were inversely related with waist circumference.

Suggestions of multiplicative interaction were seen between BMI categories and Common Brazilian meal dietary pattern (P3) for metabolic syndrome (p = 0.024), Common Brazilian fast foods/full-fat dairy/desserts dietary pattern (P2) for incident diabetes (p = 0.10) and diet/light foods and beverages/low fat dairy pattern for newly diagnosed diabetes (p = 0.157). In stratified analyses the associations between the Common Brazilian meal dietary pattern (P3) and metabolic syndrome were stronger in normal BMI participants than in the overweight or obese. The Common Brazilian fast foods/full-fat dairy/desserts was not associated with newly diagnosed diabetes in normal BMI participants, but was so associated in overweight and obese participants (p = 0.004).

## Discussion

In this large cohort of free-living middle-aged and older Brazilian men and women, after controlling for potential confounders, we observed that greater adherence to the Common Brazilian meal pattern (P3: white rice, beans, beer, processed and fresh meats), was associated with higher frequencies of newly diagnosed diabetes, metabolic syndrome and all of its components, except HDL-C. In contrast, those with greater intake of a Common Brazilian fast foods/full fat dairy/milk based desserts pattern (P2) presented less diabetes (although higher waist circumference). An inverse association was also seen between the DASH diet pattern and the metabolic syndrome, blood pressure and waist circumference. Those who adhered more to the diet, light foods and beverages/low fat dairy pattern (P4) had greater prevalences of both outcomes, and higher fasting glucose, HDL-C, waist circumference (among men) and lower blood pressure. Finally, the vegetables/fruit dietary pattern (P1) did not protect against metabolic syndrome and newly diagnosed diabetes but was associated with lower waist circumference.

Our findings concerning the Common Brazilian meal pattern merit discussion. Greater consumption of beans, a typical Brazilian food with important nutrients (fiber, potassium), when combined with white rice, processed and fresh meats, and beer, was not related to a generally better metabolic status. Similar findings were observed in the multi-ethnic study of atherosclerosis (MESA), where “Beans, tomatoes, and refined grains” pattern was associated with greater risk of type 2 diabetes [31]. Three additional US cohorts (Nurses’ Health Study, Nurses’ Health Study 2, and Health Professionals’ Follow-up Study) found that similar dietary patterns, including regular consumption of refined grains, red and processed meats, and sugar-sweetened beverages including fruits juices were associated with increased risk of diabetes [32]. Further, in a northern German cohort, a pattern characterized by high intake of potatoes, various vegetables, beef, pork, processed meat, fats, sauce and bouillon, reflecting a ‘traditional German diet’, was positively associated with metabolic syndrome [33].

Possible mechanistic factors linking empirically derived dietary patterns with measures of metabolic dysfunction have been previously described [34, 35], including associations with some markers of inflammation and endothelial activation. In the MESA Study, a “fats, oils, processed meats, fried potatoes, salty snacks, and desserts” pattern, considered a Westernized dietary pattern, was positively associated with C-reactive protein, interleukin 6, and homocysteine. The “beans, tomatoes, and refined grains” pattern was positively related to soluble intercellular adhesion molecule-1 [34]. A systematic review found positive association between Westernized dietary patterns (higher intakes of processed meats, sweets, fried foods, and refined grains) and inflammation molecules and atherogenic promoters [35, 36].

That greater adherence to the Common Brazilian fast foods/full fat dairy/milk based desserts pattern-P2 was associated with a lower frequency of newly diagnosed diabetes and a DASH diet pattern, as defined a priori, with a lower prevalence of metabolic syndrome might be explained, at least in part, by the dairy products present in these patterns. Dairy products are dense in nutrients, including dietary saturated fatty acids present in full-fat dairy products. It has been postulated that the impact of these saturated fatty acids on metabolic risk may be influenced by the food and nutrient matrix involved with dairy intake, including micronutrients such as calcium, potassium, and magnesium, which may contribute to lower the risk of newly diagnosed diabetes and cardiovascular diseases [37]. In our recent finding from the ELSA-Brasil study, the only nutrient that materially changed the associations of greater dairy intake with lesser frequency of metabolic syndrome was saturated fatty acids from dairy products [22]. Similarly, a population-based prospective cohort study in Sweden found that high intake of full-fat dairy products was associated with decreased incidence of diabetes, compared to low-fat dairy products, whereas the consumption of both low- and high-fat meats was associated with increased incidence [38]. Addition of full fat dairy as a covariate in our adjusted model in fact decreased somewhat the inverse association found, suggesting that full fat dairy may mediate, in part, this association.

It is not clear how dietary fat content and food sources of fat may modulate the effect of saturated fatty acids on risk of diabetes and metabolic syndrome. The major food sources of saturated fatty acid are of animal origin, including meat and dairy products. Ericson et al. [38] observed a decreased incidence of diabetes at high intake of high-fat dairy products but not of low-fat dairy products, and meat intake was associated with increased risk independently of fat content. Analyzing saturated fatty acids with shorter chain lengths (4:0–10:0), mainly found in dairy products, the study indicated protective associations with diabetes. Dairy products are also better sources of lauric acid (12:0) and myristic acid (14:0). In contrast, palmitic acid (16:0) and stearic acid (18:0), which are abundant in both dairy foods and meat, fish, and eggs, showed null associations with diabetes [38]. In our previous ELSA analysis, we found that myristic acid could explain the association between dairy product consumption and glycemia, insulin, and glycated hemoglobin concentrations [23]. A Dutch study with 12 years of follow-up found a lower ischemic heart disease risk with a higher intake of saturated fatty acids, and that this association was apparently mainly driven by short- to medium-chain saturated fatty, myristic acid, the sum of pentadecylic and margaric acids, and saturated fatty acids from dairy sources including butter, cheese, and milk and milk products [39]. A recent meta-analysis relating diabetes with consumption of butter, the dairy product highest in fat content, found that in four cohort studies (201,628 participants and 23,954 incident diabetes cases) butter consumption was associated with lower incidence of type 2 diabetes, with 4% lower risk per 14 g daily intake (RR = 0.96; 95% CI 0.93,0.99) [40].

In the present study we found that blood pressure was lower with increased adherence to diet, light foods and beverages/low fat dairy (P4) and DASH diet patterns, which both include low fat dairy in their composition. We previously observed in ELSA a significant, inverse, graded dose–response association between total dairy intake and adjusted systolic and diastolic blood pressure [24]. In addition to the saturated fatty acids present in dairy, other nutritional components may be involved in the reduction of the metabolic syndrome. Milk proteins have an angiotensin-converting enzyme-inhibitory effect and the inhibition of the renin angiotensin system in adipocytes can potentially reduce hypertension [41]. Calcium reduces blood pressure by modulation of smooth muscle reactivity and vitamin D may reduce dyslipidemia and improve blood pressure through maintenance of calcium homeostasis, stimulation of insulin production and release, and regulation of the renin–angiotensin–aldosterone system [40]. In contrast, a recent Mendelian randomization study using a lactase persistence genotype as an instrumental variable found that while this genotype was associated with greater dairy consumption, it showed no association with blood pressure [42].

Our findings of increased frequencies of newly diagnosed diabetes and the metabolic syndrome among those with greater adherence to the dietary pattern diet, light foods and beverages/low fat dairy (P4) might be explained by reverse causality, since a presumed knowledge of increased risk of diabetes could have led some participants to this pattern of “dieting”, or due to residual confounding, as previously suggested [43, 44]. Two additional interpretations are worth considering. First, habitual consumption of artificially sweeteners may increase the risk of diabetes and the metabolic syndrome [45–47], perhaps by alterations in the intestinal microbiota [48] or by imbalance of sweetness and caloric content (simulating a fasting state), which may trigger sensory and behavioral responses to increase caloric consumption [49]. Second, very low dietary fat intake (<15%) has been related with a higher incidence of metabolic syndrome [50].

Our findings that greater adherence to the vegetables/fruits (P1) dietary pattern did not associate with lesser frequencies of newly diagnosed diabetes and the metabolic syndrome, and was only associated with slightly lower waist circumferences are puzzling. Interestingly, in the PREDIMED trial, a significant inverse association for fiber and fruits with risk of CVD was seen only in minimally adjusted models, but not in fully adjusted models [51].

It is reassuring that the dietary patterns here derived were similar to those generated using a different methodology [52]. In the previous analyses consumption of fast-foods (and other unhealthy foods) were mixed with beans, fruits and vegetables, and most likely decreased the potential nutritional benefits of the latter foods. In this regard, it is important to highlight the emphasis on natural rather than processed foods given in the new Brazilian Dietary Guideline for the Brazilian population.

A major strength of this study is the direct measurements of the outcomes, including measurements of the cardiometabolic risk factors and detection of diabetes by a standardized 75-g oral-glucose-tolerance test and glycated hemoglobin. However, this study has some limitations. We measured dietary intake with a FFQ, a typical choice in large epidemiologic studies. This method is subject to random and systematic errors, although this limitation would most likely have resulted in a nondifferential misclassification with respect to outcomes and a likely underestimation of associations. Another limitation is that the factor analyses used to identify the dietary patterns depend on several decisions made by the researcher, such as number of factors to be retained. Furthermore, we used cross-sectional data to identify the association of dietary patterns with the metabolic syndrome and diabetes, and because of the observational nature of our study, it is possible that our findings result from reverse causality or residual confounding, the latter despite the fact that we extensively adjusted for other dietary variables and risk factors. It is also possible that some of the participants for whom we ascertained newly diagnosed diabetes reported a higher adherence to the vegetable/fruits and to the diet/light food patterns in response to their perceived propensity to develop diabetes (e.g. family history, knowledge of intermediate hyperglycemia). Although the exclusions we made aimed to prevent some of these potential biases, they may also introduce selectin bias. However, previously reported comparisons suggest that this is unlikely [23].

In conclusion, the inverse associations found for the dietary pattern characterizing Brazilian fast foods and desserts, typically containing dairy products, with newly diagnosed diabetes, and for the DASH diet with metabolic syndrome, support previously demonstrated beneficial effects of dairy products in metabolism. The positive association with metabolic syndrome and newly diagnosed diabetes found for the pattern characterizing a typical Brazilian meal deserves further investigation, particularly since it is frequently accompanied by processed meat.

## Additional file

**Additional file 1: Table S1.** Means of servings/day (sv/d) for food groups according to quintiles of adherence to each dietary pattern. ELSA-Brasil 2008–2010 (n=10,010). **Table S2.** Characteristics of Brazilian participants without previously diagnosed diabetes by categories of high adherence to dietary patterns: ELSA-Brasil 2008–2010 (n=10,010).
