# Supplementary material for: Brazilian dietary patterns and the dietary approaches to stop hypertension (DASH) diet-relationship with metabolic syndrome and newly diagnosed diabetes in the ELSA-Brasil study
Source: Diabetol Metab Syndr. 2017 Feb 13;9:13. doi: 10.1186/s13098-017-0211-7 (PMC5307839; doi:10.1186/s13098-017-0211-7)
Supplement: Supplementary file 1 — Additional file 1: Table S1. Means of servings/day (sv/d) for food groups according to quintiles of adherence to each dietary pattern. ELSA-Brasil 2008–2010 (n=10,010). Table S2. Characteristics of Brazilian participants without previously diagnosed diabetes by categories of high adherence to dietary patterns: ELSA-Brasil 2008–2010 (n=10,010). [file 13098_2017_211_MOESM1_ESM.docx]

Supplementary table 1: Means of servings/day (sv/d) for food groups according to quintiles of adherence to each dietary pattern. ELSA-Brasil 2008–2010 (n=10,010).

| **Mean ± SE of servings per day for food groups across quintiles of adherence to each dietary pattern** | | | |
| --- | --- | --- | --- |
|  | **Quintile 1 – low adherence** | **Quintile 3 – mid adherence** | **Quintile 5 – high adherence** |
| **P1 - Vegetables/fruits** |  |  |  |
| Total vegetables, sv/d | 5.6 ± 0.11 | 10.3 ± 0.11 | 21.5 ± 0.11 |
| Total fruits, sv/d | **1.8 ± 0.05** | **3.8 ± 0.05** | **7.5 ± 0.05** |
| Whole grains, sv/d | **0.7 ± 0.05** | **1.5 ± 0.05** | **2.5 ± 0.05** |
| **P2 - Common Brazilian fast foods/full fat dairy/desserts** | |  |  |
| Refined grains, sv/d | 6.4 ± 0.11 | 7.0 ± 0.11 | 9.5 ± 0.11 |
| Full fat dairy, sv/d | **0.9 ± 0.04** | **1.8 ± 0.04** | **3.4 ± 0.04** |
| **P3 - Common Brazilian meal** |  |  |  |
| Whole grains, sv/d | **3.4 ± 0.05** | **1.2 ± 0.05** | **0.5 ± 0.05** |
| Refined grains, sv/d | **5.6 ± 0.10** | **7.0 ± 0.10** | **10.4 ± 0.10** |
| Red and processed meat, sv/d | 1.1 ± 0.03 | 1.6 ± 0.03 | 2.7 ± 0.03 |
| **P4 - Diet, light foods and beverages/ low fat dairy** | |  |  |
| Whole grains, sv/d | **1.1 ± 0.05** | **1.4 ± 0.05** | **2.0 ± 0.05** |
| Refined grains, sv/d | 10.1 ± 0.11 | 7.1 ± 0.11 | 6.0 ± 0.11 |
| Artificial sweetened beverages, sv/d | **0.1 ± 0.04** | **0.9 ± 0.04** | **3.0 ± 0.04** |

Supplementary table 2: Characteristics of Brazilian participants without previously diagnosed diabetes by categories of high adherence to dietary patterns: ELSA-Brasil 2008–2010 (n=10,010).

|  |  | **Adherence to different dietary patterns (in quintiles)** | | |
| --- | --- | --- | --- | --- |
|  |  | **Quintile 1 – low adherence** | **Quintile 3 – mid adherence** | **Quintile 5 – high adherence** |
| **Characteristics** | **Dietary Patterns** |  | **N (%) or Mean ±SD** |  |
| Age, y | Vegetables/Fruits | 48.6 ± 8.2 | 50.8 ± 8.7 | 52.8 ± 8.7 |
|  | Common Brazilian fast food/Full fat dairy/desserts | 53.8 ± 8.4 | 50.7 ± 8.7 | 48.2 ± 8.4 |
|  | Common Brazilian meal | 52.6 ± 9.1 | 50.8 ± 8.7 | 49.4 ± 7.7 |
|  | Diet, light foods and beverages/ low fat dairy | 50.8 ± 8.4 | 50.6 ± 8.8 | 51.2 ± 8.7 |
|  | DASH Diet | 47.9 ± 7.7 | 50.3 ± 8.4 | 54.1 ± 8.9 |
| Sex, F, n (%) | Vegetables/Fruits | 817 (42.0) | 1091 (56.1) | 1154 (59.3) |
|  | Common Brazilian fast food/Full fat dairy/desserts | 1192 (61.3) | 1112 (57.2) | 833 (42.8) |
|  | Common Brazilian meal | 1326 (68.2) | 1158 (59.5) | 548 (28.2) |
|  | Diet, light foods and beverages/ low fat dairy | 979 (50.3) | 1028 (52.9) | 1154 (59.3) |
|  | DASH Diet | 946 (54.2) | 1266 (55.3) | 1047 (57.1) |
| Self-identified skin color/race category, n (%) |  |  |  |  |
| White | Vegetables/Fruits | 1021 (53.0) | 1032 (53.4) | 888 (46.3) |
|  | Common Brazilian fast food/Full fat dairy/desserts | 705 (36.6) | 1058 (55.0) | 1134 (58.8) |
|  | Common Brazilian meal | 1144 (59.7) | 1043 (53.9) | 982 (50.9) |
|  | Diet, light foods and beverages/ low fat dairy | 633 (32.8) | 1009 (52.4) | 1318 (68.7) |
|  | DASH Diet | 814 (47.0) | 1193 (52.6) | 1006 (55.6) |
| Brown (pardo or mixed) | Vegetables/Fruits | 594 (30.8) | 557 (28.8) | 572 (29.9) |
|  | Common Brazilian fast food/Full fat dairy/desserts | 698 (36.3) | 530 (27.5) | 482 (25.0) |
|  | Common Brazilian meal | 471 (24.6) | 536 (27.7) | 692 (36.1) |
|  | Diet, light foods and beverages/ low fat dairy | 754 (39.1) | 559 (29.0) | 386 (20.1) |
|  | DASH Diet | 532 (30.5) | 648 (28.6) | 505 (27.9) |
| Educational level, University degree, n (%) | Vegetables/Fruits | 1135 (58.4) | 1082 (55.6) | 898 (46.2) |
|  | Common Brazilian fast food/Full fat dairy/desserts | 826 (42.5) | 1137 (58.5) | 1059 (54.4) |
|  | Common Brazilian meal | 1410 (72.5) | 1116 (57.4) | 551 (28.3) |
|  | Diet, light foods and beverages/ low fat dairy | 509 (26.2) | 1050 (54.0) | 1578 (81.1) |
|  | DASH Diet | 720 (41.3) | 1277 (55.8) | 1166 (63.6) |
| Family history of diabetes, n (%) | Vegetables/Fruits | 686 (35.3) | 687 (35.3) | 736 (37.8) |
|  | Common Brazilian fast food/Full fat dairy/desserts | 783 (40.3) | 658 (33.8) | 714 (36.7) |
|  | Common Brazilian meal | 653 (33.6) | 719 (37.0) | 737 (37.9) |
|  | Diet, light foods and beverages/ low fat dairy | 691 (35.5) | 685 (35.2) | 720 (37.0) |
|  | DASH Diet | 643 (36.9) | 842 (36.8) | 680 (37.1) |
| Current smoker, n (%) | Vegetables/Fruits | 314 (16.1) | 247 (12.7) | 202 (10.4) |
|  | Common Brazilian fast food/Full fat dairy/desserts | 251 (12.9) | 228 (11.7) | 280 (14.4) |
|  | Common Brazilian meal | 106 (5.4) | 234 (12.0) | 436 (22.4) |
|  | Diet, light foods and beverages/ low fat dairy | 238 (25.9) | 260 (13.4) | 221 (11.4) |
|  | DASH Diet | 331 (19.0) | 287 (12.5) | 116 (6.3) |
| Alcohol intake, g ethanol/d | Vegetables/Fruits | 94.7 ± 163.6 | 59.8 ± 112.1 | 54.0 ± 106.5 |
|  | Common Brazilian fast food/Full fat dairy/desserts | 72.7 ± 152.4 | 63.3 ± 122.7 | 70.1 ± 122.7 |
|  | Common Brazilian meal | 36.5 ± 70.4 | 52.4 ± 91.1 | 134.4 ± 206.8 |
|  | Diet, light foods and beverages/ low fat dairy | 32.2 ± 76.0 | 67.2 ± 127.0 | 101.9 ± 155.8 |
|  | DASH Diet | 78.7 ± 161.3 | 70.9 ± 127.4 | 47.7 ± 88.4 |
| Physical activity (moderate and vigorous), | Vegetables/Fruits | 337.7 ± 781 | 419.6 ± 866 | 552.2 ± 1154 |
| MET-min/wk | Common Brazilian fast food/Full fat dairy/desserts | 390.2 ± 947 | 410.9 ± 881 | 455.9 ± 989 |
|  | Common Brazilian meal | 585.9 ± 1054 | 364.3 ± 808 | 339.7 ± 828 |
|  | Diet, light foods and beverages/ low fat dairy | 278.8 ± 778 | 402.7 ± 859 | 661.3 ± 1144 |
|  | DASH Diet | 244.6 ±688 | 420.7 ± 900 | 650 ± 1166 |
| Nutrients, % energy |  |  |  |  |
| Total fat | Vegetables/Fruits | 30.3 ± 5.5 | 28.4 ± 4.8 | 26.7 ± 4.9 |
|  | Common Brazilian fast food/Full fat dairy/desserts | 25.2 ± 4.9 | 28.8 ± 4.9 | 31.4 ± 4.7 |
|  | Common Brazilian meal | 27.3 ± 5.3 | 28.7 ± 5.2 | 29.2 ± 5.0 |
|  | Diet, light foods and beverages/ low fat dairy | 26.1 ± 4.7 | 28.8 ± 5.1 | 30.6 ± 5.3 |
|  | DASH Diet | 31.0 ± 5.0 | 28.6 ± 4.8 | 25.6 ± 4.8 |
| Total SFAs | Vegetables/Fruits | 10.6 ± 2.9 | 9.8 ± 2.4 | 8.8 ± 2.3 |
|  | Common Brazilian fast food/Full fat dairy/desserts | 8.0 ± 2.3 | 9.9 ± 2.4 | 11.2 ± 2.4 |
|  | Common Brazilian meal | 9.8 ± 2.8 | 9.9 ± 2.6 | 9.3 ± 2.3 |
|  | Diet, light foods and beverages/ low fat dairy | 8.9 ± 2.4 | 9.8 ± 2.6 | 10.8 ± 2.7 |
|  | DASH Diet | 10.7 ± 2.6 | 9.9 ± 2.5 | 8.5 ± 2.4 |
| Protein | Vegetables/Fruits | 18.6 ± 3.6 | 18.8 ± 3.7 | 18.8 ± 3.9 |
|  | Common Brazilian fast food/Full fat dairy/desserts | 19.5 ± 4.4 | 18.8 ± 3.7 | 17.9 ± 3.3 |
|  | Common Brazilian meal | 18.4 ± 3.6 | 19.0 ± 4.0 | 18.4 ± 3.6 |
|  | Diet, light foods and beverages/ low fat dairy | 16.3 ± 2.8 | 18.6 ± 3.3 | 21.3 ± 3.8 |
|  | DASH Diet | 18.5 ± 3.7 | 18.7 ± 3.8 | 19.0 ± 3.8 |
| Carbohydrate | Vegetables/Fruits | 49.5 ± 8.0 | 53.0 ± 7.6 | 56.1 ± 8.2 |
|  | Common Brazilian fast food/Full fat dairy/desserts | 55.3 ± 8.8 | 52.5 ± 8.2 | 50.9 ± 7.7 |
|  | Common Brazilian meal | 55.9 ± 8.1 | 52.5 ± 8.3 | 50.8 ± 7.9 |
|  | Diet, light foods and beverages/ low fat dairy | 58.9 ± 6.7 | 52.7 ± 7.2 | 47.2 ± 7.8 |
|  | DASH Diet | 49.7 ± 8.0 | 52.6 ± 7.8 | 56.9 ± 7.8 |
| BMI (kg/m2) | Vegetables/Fruits | 26.5 ± 4.5 | 26.6 ± 4.6 | 26.9 ± 4.7 |
|  | Common Brazilian fast food/Full fat dairy/desserts | 26.8 ± 4.5 | 26.4 ± 4.5 | 27.0 ± 4.7 |
|  | Common Brazilian meal | 25.4 ± 4.1 | 26.7 ± 4.4 | 27.6 ± 4.8 |
|  | Diet, light foods and beverages/ low fat dairy | 25.9 ± 4.6 | 26.5 ± 4.5 | 27.5 ± 4.7 |
|  | DASH Diet | 27.0 ± 4.8 | 26.7 ± 4.5 | 26.2 ± 4.3 |
